# Are solid state nuclear track detectors truly integrated devices?—experimental evidence in radon measurements

**DOI:** 10.1093/rpd/ncae060

**Published:** 2024-03-21

**Authors:** Jaroslaw M Wasikiewicz

**Affiliations:** United Kingdom Health Security Agency, RCE, Chilton OX11 0RQ, United Kingdom

## Abstract

Passive diffusion radon monitoring devices were exposed to a range of radon concentrations and their variations. The experimental results, backed up by the statistical analysis, showed that the radon concentration had virtually no effect on the final integrated exposure results, with the exception for the very low concentration. Therefore, it was proven that such devices are truly integrated exposure devices.

## Introduction

Passive detectors are used for measurements of radon concentration in households and workplaces. Usually, such devices are in place for three months and the recorded value is then extrapolated to the annual exposure. Annual radon exposure is used to evaluate the need for remediation action for the household or to ensure the legal requirements of health and safety for workplaces are met.

The passive solid state nuclear track radon diffusion detectors (SSNTD) with a poly-allyl-diglycol carbonate (PADC) sensing element inside are integrated exposure detectors. It means that the measurement results are the average of all recorded radon concentrations during the exposure period. This is a well-known and acknowledged property of SSNTDs within the scientific community and as highlighted by Durrani, together with an ability to record exposure over long periods of time are their greatest strengths^(1)^. However, some radon professionals question whether different concentrations of radon will impact the outcome of measurements for such devices. A peculiar case is the intercomparison exercise^(2)^, at which detectors are usually exposed to high radon concentrations. Therefore, the recurring comment of participants is that, for example, integrated exposure of 400 kBq h m^−3^ over a few days at thousands of Bq m^−3^ is not the same as over 3 months at tens of Bq m^−3^. Although, such high radon concentrations may be falsely considered as ‘non-standard’ conditions, but our experience of households measurements show it can vary from 0 up to 25 000 Bq m^−3^.

Limited study on the subject exists, and it is mostly aimed to compare SSNTDs with electronic, active devices. Even though they provide valuable data, e.g. either good or bad correlation between instruments, they are usually limited to exposure only at one or two radon concentrations^(3, 4)^.

The aim of this study was to investigate, providing experimental evidence, whether the radon concentration can have an impact on the final results of integrated exposure measurements.

## Materials and methods

### Passive detectors

Nuclear solid state track detectors consisting of PADC sensing elements enclosed in a diffusion chamber were used in this study. After manufacture at Mi-Net Technology Ltd UK, sheets of plastic were kept for 1 month in radon proof pouches under ambient air to avoid a sudden drop in sensitivity^(5)^. Then, 1-mm thick sheets of PADC were cut and assembled into a standard UKHSA radon detector housing. After the exposure to radon, the latent alpha tracks in PADC were revealed by chemical etching with NaOH (5 M) at a temperature of 75°C for 18 h.

### Exposures

Groups of 15 passive detectors were exposed to various radon concentrations to reach equal integrated exposures of around 37 kBq h m ^−3^.

Low activities were provided by granite stones (label G in Table 1) enclosed in a 0.30-m^3^ steel box were monitored with AlphaGUARD DF2000 from Saphymo GmbH, Germany. Mid-range radon activities were sourced from flow-through source (FF) in the UKHSA 43-m^3^ radon chamber and were monitored by Alphaguard EF 2272 from Saphymo GmbH and ATMOS 12DPX system (pulse ionisation chamber technology) manufactured by Gammadata Instrument AB, Sweden. Finally, a radium painted watch dial (RWD) enclosed in a 0.28-m^3^ steel barrel was the source of high radon activities, which were monitored with AlphaGUARD EF0742 from Genitron Instruments GmbH, Germany. All monitoring instruments were calibrated against a primary source traceable to CHUV, Switzerland. The measurement error between all monitoring devices was <5%. Environmental parameters such as temperature, humidity and air pressure were monitored, but not controlled during exposures. Exposures were performed either at a single radon concentration—Series 1–7 or a mixture—series M1–M6 of radon concentrations.

**Table 1:** Summary of groups exposure data. G—granite blocks, FF—flow through radon source, RWD—radium watch dial; AG—AlphaGUARD, A—ATMOS.

Code	Integrated exposure [Bq h m^−3^]	Average ^222^Rn concentration [Bq m^−3^]	Exposure time [dd:hh:mm]	Radon source	Monitoring device	Pressure [Pa]	Humidity [%]	Temperature [°C]
1	34 000 ± 4087	18 ± 2	78:22:25	G	AG	1011 ± 1	44.9 ± 3.3	21.3 ± 3.4
2	37 057 ± 5045	79 ± 11	19:07:00	G	AG	997 ± 1	48.3 ± 0.2	26.3 ± 0.2
3	37 306 ± 4642	122 ± 16	12:16:20	G	AG	1001 ± 5	53.3 ± 1.0	25.9 ± 0.6
4	37 553 ± 1947	1459 ± 76	00:25:44	FF	AG	1000 ± 3	38.4 ± 0.4	21.5 ± 0.2
5	37 069 ± 1398	4763 ± 182	00:08:47	FF	A	1009 ± 1	37.2 ± 0.5	24.2 ± 1.0
6	37 056 ± 1307	8551 ± 301	00:04:20	FF	A	1004 ± 1	36.4 ± 0.2	26.6 ± 0.1
7	37 603 ± 940	85 504 ± 2080	00:00:27	RWD	AG	998 ± 1	35.8 ± 0.1	28.4 ± 0.1
M1	19 261 ± 768 + 22 846 ± 556	4315 ± 172 + 67 072 ± 1684	00:03:28 + 00:00:20	FF + RWD	A + AG	1012 ± 1	29.4 ± 0.6	23.4 ± 1.2
M2	29 819 ± 735 + 7576 ± 295	69 888 ± 1764 + 439 ± 170	00:00:25 + 00:01:44	RWD + FF	AG + A	1012 ± 1	29.6 ± 1.0	23.8 ± 1.8
M3	20 978 ± 784 + 16 156 ± 418	7017 ± 263 + 58 112 ± 1476	00:02:59 + 00:00:17	FF + RWD	A + AG	1015 ± 1	29.1 ± 0.8	24.5 ± 0.6
M4	24 576 ± 624 + 11 692 ± 440	61 440 ± 1560 + 6963 ± 264	00:00:24 + 00:01:40	RWD + FF	AG + A	1015 ± 1	29.1 ± 0.8	24.8 ± 1.6
M5	20 037 ± 762 + 17 465 ± 2335	4790 ± 184 + 91 ± 12	00:04:09 + 8:00:00	FF + G	A + AG	987 ± 2	48.6 ± 4.3	23.2 ± 2.4
M6	31 728 ± 1440 + 5233 ± 202	110 + 4764	11:23:50 + 00:01:06	G + FF	A + AG	996 ± 1	57.7 ± 4.4	23.0 ± 2.4
EQ1	35 150 ± 1966	4155 ± 232	00:08:28	FF	A	983 ± 1	35.3 ± 0.1	19.4 ± 0.1
EQ2	40 315 ± 1426	4743 ± 168	00:08:30	FF	A	993 ± 1	34.1 ± 0.2	20.4 ± 0.1
EQ3	37 807 ± 1357	5388 ± 194	00:07:01	FF	A	1012 ± 1	33.6 ± 0.2	22.0 ± 0.3

The equilibrium factor is the ratio of the equilibrium equivalent concentration of radon to the actual radon concentration. Exposures at different equilibrium factors (EQ1–EQ3 series) were achieved by varying the size and the concentration of carnauba wax particles generated by aerosol generator pumped into the radon chamber.

### Analysis of passive detectors

Images of etched detectors were recorded by a Nikon LS5000ED, Japan, slide scanner at the resolution of 4000 dpi. Subsequently, images were analysed and tracks and/or area covered by tracks calculated using the image analysis software. The software was supplied by Synopsis Ltd, UK, to a specification provided by NRPB (UKHSA predecessor). Full details of the first version of the software were described by Steele *et al*.^(6)^.

### Statistical analysis

Statistical analysis was performed using Data Analysis Tools included in Microsoft Excel 365 software version 2002 (build 12527.21912). Exposure group comparisons were based on the analysis of variance (one-way ANOVA test) and post hoc Bonferroni test at the significance level *α* = 0.05.

## Results and discussion

**Figure 1:**
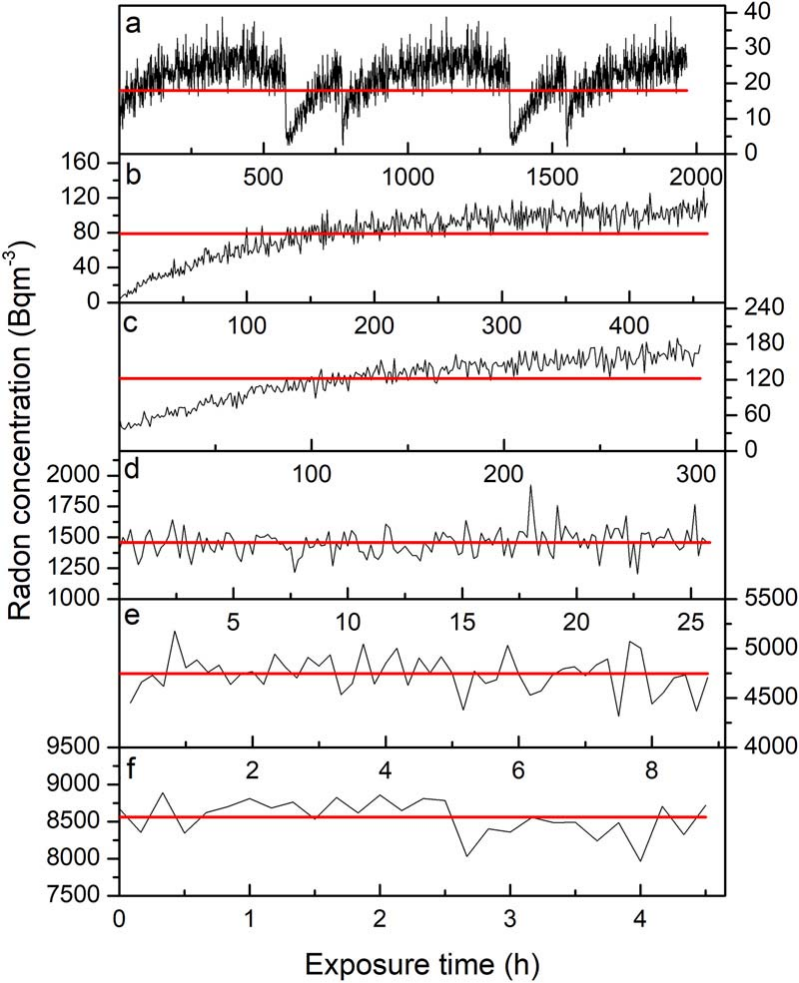
Reference radon concentration during passive detectors exposures. Horizontal lines represent the average values.

Examples of monitoring data of reference radon exposures for selected measurement groups were shown in Figure 1. A few drops in radon concentration for the lowest value (a) were observed, which was because of the controlled release of radon gas to prevent excessive concentration build up. At higher concentrations, the experimental set-ups were assembled with the radon source and passive detectors placed together and then sealed. Hence, a small delay to reach the stable radon concentration was noticed (b, c). Other examples show cases where passive detectors were placed in containments with pre-equilibrated, stable radon concentrations and where such actions have not disturbed the radon environments. The horizontal line was drawn to indicate the average radon concentration values.

Monitoring data for all exposures were collected in Table 1. The target integrated exposure was aimed to be around 40 kBq h m^−3^, which approximates the average annual radon exposure in the UK households ~20 Bq m^−3^, measured for 3 months^(7)^. Although, the exact value of the integrated exposure for all set-ups was very hard to achieve, the final numbers were within quite good agreement, even though the exposure time ranged from minutes to months. Some, small differences in environmental conditions, i.e. atmospheric pressure, humidity and temperature, were observed, but such low values should have only a negligible, if any effect on the final measurements^(8)^.

The average exposures of measured SSNTD groups were collected in Table 2. These results were normalised against reference radon concentrations measured by calibrated monitoring system (as in Table 1). Most normalised values showed a good agreement with the reference value except for the Group 1—at the lowest radon concentration. This was most likely because of the fact that monitoring equipment was at its lower detection limit and hence carried a relatively high measurement uncertainty. It is also known, that at very low radon concentrations such equipment tends to misread the real values as it cannot respond quickly enough to record small, but fast changes. Also, it was demonstrated that even detectors of the same model can significantly deviate from the reference value at low radon concentrations^(9)^.

**Table 2:** Average and normalised exposures recorded by passive detectors.

Group code	Average measured exposure [kBq h m^−3^]	Average measured STD [kBq h m^−3^]	Normalised avg. exposure
1	26 563	3303	0.780
2	34 666	2772	0.935
3	36 867	3073	0.988
4	36 553	2704	0.973
5	38 133	2445	1.029
6	36 467	2305	0.984
7	36 000	4816	0.957
M1	40 268	4019	0.973
M2	37 066	8891	1.010
M3	36 865	6843	0.993
M4	37 782	6068	1.042
M5	34 867	2295	0.930
M6	32 867	2100	0.890
EQ1	35 051	1949	0.997
EQ2	41 620	6048	1.032
EQ3	38 129	3210	1.009

In order to perform statistical analysis of results of radon concentration on the final integrated exposure measurement, the statistical group analysis using one-way ANOVA (variance analysis) was run. The data used in the statistical analysis have been collected in Tables 3a, 4a and 5a.

**Table 3a:** Summary of ANOVA test of passive detectors exposed at a single radon concentration.

Group code	Count	Sum	Average	Variance
1	15	10.993	0.734	0.009
2	14	13.250	0.946	0.005
3	15	14.823	0.988	0.007
4	15	14.593	0.973	0.006
5	15	15.431	1.029	0.005
6	15	14.761	0.984	0.004
7	15	14.361	0.957	0.018

**Table 3b:** ANOVA test results of passive detectors exposed at a single radon concentration.

Source of variation	SS	df	MS	*F*	*p* value	*F* crit
Between groups	0.845	6	0.141	18.638	2.62E−14	2.194
Within groups	0.733	97	0.008			
Total	1.578	103				

**Table 3c:** Post hoc Bonferroni test for passive detectors exposed at a single radon concentration.

Groups	*p* value	Significant
1v2	1.74E−07	Yes
1v3	1.82E−08	Yes
1v4	2.05E−08	Yes
1v5	1.40E−10	Yes
1v6	2.98E−09	Yes
1v7	1.09E−05	Yes
2v3	1.56E−01	No
2v4	3.27E−01	No
2v5	2.97E−03	No
2v6	1.35E−01	No
2v7	7.84E−01	No
3v4	6.03E−01	No
3v5	1.62E−01	No
3v6	8.82E−01	No
3v7	4.55E−01	No
4v5	4.12E−02	No
4v6	6.62E−01	No
4v7	6.97E−01	No
5v6	7.63E−02	No
5v7	7.45E−02	No
6v7	4.88E−01	No
Post-test Bonferroni at *α* = 0.05	0.0024	

**Table 4a:** Summary of ANOVA test of passive detectors exposed at two different radon concentrations.

Group code	Count	Sum	Average	Variance
M1	15	14.592	0.974	0.009
M2	14	13.593	0.971	0.038
M3	13	12.906	0.993	0.034
M4	15	15.626	1.042	0.028
M5	14	12.826	0.916	0.005
M6	15	13.490	0.899	0.003

**Table 4b:** ANOVA test results of passive detectors exposed at two different radon concentrations.

Source of variation	SS	df	MS	*F*	*p* value	*F* crit
Between groups	0.198	5	0.040	2.048	0.081	2.329
Within groups	1.546	80	0.019			
Total	1.744	85				

**Table 5a:** Summary of ANOVA test of passive detectors exposed at various equilibrium factors.

Group code	Count	Sum	Average	Variance
EQ 0.1	15	14.958	0.997	0.003
EQ 0.4	15	15.486	1.032	0.023
EQ 0.9	15	15.128	1.009	0.007

The null hypothesis raised was that in studied groups of detectors exposed to different radon concentrations there was not a difference in the variance of normalised, average measured exposure between these groups. On the other hand, the alternative hypothesis was that in studied groups of detectors exposed to different radon concentrations there was a difference in the variance of normalised, average measured exposure between these groups. Based on the results, it was concluded that at *α* = 0.05, 6 degrees of freedom between groups and 97 degrees of freedom within groups and p-value being significantly lower than *α* (*F*(6, 97) = 18.64, *p* < 0.001), there was a significant difference between mean values of recorded integrated exposures between seven tested groups of detectors exposed to radon concentrations (Table 3b), and hence the alternative hypothesis must be accepted.

Although the test showed significant difference between mean values between groups, it has not indicated which group or groups are outliers. To establish it, a consecutive post hoc Bonferroni correction test^(10)^ was run. Second reason to apply Bonferroni correction was to lower the possibility of occurrence of false positive results in is a multiple comparison of independent statistical tests being performed simultaneously. Since, while a given alpha value  *α* may be appropriate for each individual comparison, it is not for the set of all comparisons. The post hoc analysis at *α* = 0.05 revealed that variance in the mean exposure of Group 1 was significantly different compared with other groups and that there was not a significant difference between all other groups (Table 3c) This was in line with the general overview of results described above.

Multi-variance analysis test was also run on groups exposed to consecutive, different radon concentrations, i.e. exposure at low concentration followed by exposure at high one or in any other possible combinations. The same null hypothesis as before that: in studied groups of detectors exposed to different radon concentrations there was no difference in the variance of normalised, average measured exposure, was assessed. The results revealed that the null hypothesis must be accepted and that there was no significant difference in variance of the mean exposure values between all groups *F*(5, 80) = 20.5, *p* = 0.08 at *α* = 0.05, 5 degrees of freedom between groups and 80 degrees of freedom within groups (Table 4b).

Finally, the ANOVA test was run on groups exposed to radon with different equilibrium factor values. Like in the case of mixed exposures, there was no significant difference in variance of mean average values of integrated exposure *F*(2, 42) = 0.44, *p* = 0.65, at *a* = 0.05, with 2 degrees of freedom between groups and 42 degrees of freedom within groups (Table 5b).

**Table 5b:** ANOVA test results of passive detectors exposed at various equilibrium factors.

Source of variation	SS	df	MS	*F*	*p* value	*F* crit
Between groups	0.010	2	0.005	0.443	0.645	3.220
Within groups	0.459	42	0.011			
Total	0.469	44				

## Conclusions

The experimental evidence showed that there was no significant difference in recorded integrated exposure regardless of the radon concentration or equilibrium factor. The only exception was for the lowest radon concentration, but it may be because of the low limit of detection of monitoring devices rather than measurements with passive detectors. Therefore, it has been proven that diffusion chamber solid state nuclear track passive radon detectors are truly integrated exposure devices. This means whatever radon concentration, or its mixture passive detectors are exposed to, they will record the total (integrated) value of radon concentration, to which they were exposed.
